# Tailoring the Shape‐Memory Performance of 2D and 3D Fabricated Semi‐Crystalline PCL Networks Via Optimal Crosslinking

**DOI:** 10.1002/marc.202500631

**Published:** 2025-10-19

**Authors:** Lorenzo Bonetti, Daniele Natali, Stefano Pandini, Massimo Messori, Maurizio Toselli, Giulia Scalet

**Affiliations:** ^1^ Department of Civil Engineering and Architecture University of Pavia Pavia Italy; ^2^ Department of Industrial Chemistry "Toso Montanari" University of Bologna Bologna Italy; ^3^ Department of Mechanical and Industrial Engineering University of Brescia Brescia Italy; ^4^ Department of Applied Science and Technology Politecnico Di Torino Torino Italy

**Keywords:** 3D printing, PCL, photo‐crosslinking, semi‐crystalline polymer networks, shape‐memory polymers

## Abstract

Photo‐crosslinking is a fast and efficient approach to obtain chemically crosslinked semi‐crystalline networks featuring both one‐way and two‐way shape‐memory effect. However, the effect of photo‐crosslinking parameters and fabrication method on the physical, thermo‐mechanical, and shape‐memory properties of these networks still has to be investigated. This paper aims to fill this gap, specifically focusing on semi‐crystalline polycaprolactone (PCL) networks. In detail, the influence of key photo‐crosslinking parameters ‐crosslinking temperature and UV light intensity‐ as well as the fabrication method ‐2D vs. 3D‐ were investigated. As a general trend, crosslinking above the melting temperature of PCL and selecting a high UV light intensity yielded structures with superior performance, also displaying stress‐free shape‐memory behavior. Conversely, crosslinking below the crystallization temperature of PCL and selecting a low UV light intensity led to reduced performance and absence of stress‐free actuation. To address this limitation, a post‐treatment involving additional UV exposure was introduced, which significantly improved overall performance, particularly enhancing the two‐way shape‐memory behavior. Interestingly, although the 3D printed samples displayed thermal properties comparable to their 2D counterparts, their shape‐memory performance was significantly reduced. Overall, these findings provide practical design guidelines for engineering 2D and 3D PCL‐based semi‐crystalline structures with tunable physical, thermal, and shape‐memory properties.

## Graphical abstract

This study investigates the impact of two key photo‐crosslinking parameters, namely the crosslinking temperature and the UV light intensity, as well as the effect of the fabrication method, i.e., 2D vs. 3D, on the physicochemical, thermal, mechanical, and shape‐memory properties of polycaprolactone (PCL)‐based semi‐crystalline polymer networks.

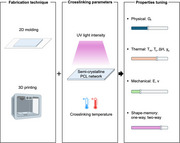

## 1 Introduction

Shape‐memory polymers (SMPs) are a class of smart materials capable of fixing a deformed ‐temporary‐ shape and subsequently recovering their original ‐permanent‐ shape upon exposure to an external stimulus, most commonly heat [1, 2]. Such a capability is commonly referred to as one‐way shape‐memory effect (SME). However, the reliance on external intervention to reprogram the temporary shape limits the application of these materials in dynamic environments. To address this limitation, two‐way SMPs have been developed, which enable reversible switching between two different configurations upon heating and cooling cycles [3, 4, 5, 6].

Among the possible classes of SMPs, semi‐crystalline polymer networks have attracted growing interest due to their ability to combine reversible phase transitions with elastic network stability, resulting in efficient and controllable one‐way and two‐way shape‐memory behaviors [7, 8, 9, 10]. In this framework, polycaprolactone (PCL) has emerged as a particularly promising candidate for designing thermo‐responsive SMPs. Its semi‐crystalline nature, low melting temperature (≈ 50–60°C), biodegradability, synthetic versatility, and the possibility to blend it with other polymers [11, 12], make it attractive for a wide range of applications, especially in the biomedical field [13, 14, 15].

In chemically crosslinked PCL‐based networks, the crystalline regions act as thermal switches that fix the temporary shape upon cooling, while the chemical net points ensure effective recovery of the original shape. This dual‐phase structure enables an effective SME [16, 17, 18]. Particularly, PCL‐based semi‐crystalline networks have been largely investigated for their thermally‐triggered two‐way SME under an applied external stress. The two‐way SME in PCL was first reported by Hong et al. [19] shortly after the identification of a reversible behavior in a semi‐crystalline polymer network based on crosslinked poly(cyclooctene) [20]. In particular, Hong and co‐workers showed that a PCL‐based shape‐memory polyurethane ‐composed of hard segments functioning as crosslinks and soft PCL segments containing both amorphous and crystallizable phases‐ exhibited noteworthy elongation under load. The mechanism was attributed to the alignment of soft‐segment chains along the loading direction, followed by oriented crystallization during cooling under constant stress [19]. Interestingly, such a reversible behavior was soon proposed also in the absence of an external stress applied, when Lendlein's group reported for the first time bidirectional SMPs capable of switching between two shapes, in stress‐free conditions, under heating/cooling cycles [21]. This pioneering work laid the foundation for the development of a plethora of different two‐way SMPs capable of self‐actuation [4, 5, 22, 23] demonstrating excellent applicability in a wide range of research fields, including biomedical devices [13], soft actuators and robots [3], and smart textiles [24].

As it was possible to understand, the obtainment of semi‐crystalline networks based on PCL relies on crosslinking, which can be achieved using various techniques, including radiation [25, 26, 27], chemical methods [11, 16, 28], and photo‐crosslinking [14, 29, 30]. In particular, photo‐crosslinked PCL‐MA networks are synthesized by modifying PCL chains with methacrylate (MA) groups (e.g., reaction with isocyanatoethyl methacrylate or glycidyl methacrylate) followed by UV‐induced radical polymerization in the presence of a photo‐initiator [14, 29, 30]. This latter strategy enables rapid and spatially‐controlled crosslinking. Moreover, the integration of photo‐crosslinkable PCL‐MA with additive manufacturing technologies has advanced the field of 4D printing, where PCL has been investigated in different approaches, among which fused filament fabrication (FFF), digital light processing (DLP), and stereolithography (SLA) [13, 14, 31].

In this regard, it is well‐known that the shape‐memory behavior and the properties of photo‐crosslinked PCL‐MA networks are significantly influenced by the conditions under which photo‐crosslinking is performed. Parameters such as UV light intensity, environmental conditions (e.g., temperature), exposure time, photo‐initiator type and concentration, can directly affect the crosslinking density, network homogeneity, and crystallinity of the final material, known to govern key shape‐memory properties [14, 15]. Despite the critical role of these parameters, systematic studies evaluating the impact of photo‐crosslinking conditions on the final shape‐memory performance of the obtained structures cannot be found in the literature. Most existing works focus on demonstrating feasibility or showcasing applications, rather than providing a detailed analysis of how processing variables influence material behavior and, most importantly, of how these can be used for tailoring material properties required by the application at hand. In addition, to the best of these authors’ knowledge, no previous works have simultaneously investigated the effect of photo‐crosslinking conditions and fabrication approaches. As a result, there is an important need for dedicated investigations that establish clear structure–property relationships in photo‐crosslinked PCL‐MA systems, especially in the context of additive manufacturing where control over printing and curing conditions can vary widely.

In this paper, we investigate the effect of two main photo‐crosslinking parameters, namely the crosslinking temperature and the UV light intensity on the behavior of photo‐crosslinked PCL‐MA networks. The photo‐crosslinking time and the photo‐initiator (type and concentration) are kept constant based on previous studies. In addition, we investigate and compare two different fabrication techniques, i.e., compression molding (used to obtain 2D samples) and 3D printing, to assess the influence of the fabrication technique on the performance of the obtained samples. A thorough physical, thermo‐mechanical, and shape‐memory characterization of the specimens is carried out to unveil the effect of the crosslinking parameters and the fabrication technique on the behavior of the obtained samples. We also investigate the possibility to post‐treat specimens obtained under low crosslinking conditions as a potential strategy to improve their performance.

Overall, this study provides useful guidelines for the optimal design of structures made from PCL‐based semi‐crystalline networks with tailored physico‐mechanical, thermal, and shape‐memory performance.

## 2 Experimental Section

### 2.1 Materials

PCL diol (𝛼,ω‐hydroxyl‐terminated PCL, *M_n_* ≈ 10 kDa), hereafter denoted as PCL10, 2‐isocyanatoethyl methacrylate (2‐IEM, 98 %), tetrahydrofuran (THF) and 2‐hydroxy‐2‐methylpropiophenone were purchased from Merck and used without any further purification step.

### 2.2 Synthesis of Methacrylated PCL

The methacrylation reaction of the hydroxyl end groups of PCL10 was carried out following a previously established protocol [30]. Briefly, dried PCL10 and 2‐IEM were placed into a glass flask at a molar ratio of 1:2.4 (2‐IEM was added in a 20% stoichiometric excess relative to the hydroxyl groups of PCL10 to ensure full conversion). Methacrylation was performed at 100°C for approximately 3–4 h under stirring in a nitrogen atmosphere. Progress of the reaction was monitored via FT‐IR spectroscopy, and considered complete when the intensity ratio of the peaks at 2930 and 2275 cm^−1^ stabilized. Lastly, unreacted 2‐IEM was removed under dynamic vacuum, and FT‐IR analysis confirmed its absence by the disappearance of the isocyanate absorption band at 2275 cm^−1^. The obtained methacrylated PCL10 (hereafter denoted as PCL10‐MA) was kept in the dark at room temperature (RT) until use. Additional information regarding the synthesis and chemical characterization of the obtained materials was available in reference [30].

### 2.3 Sample Preparation

2D and 3D specimens were prepared with two different techniques, i.e., compression molding and 3D printing. To achieve photo‐crosslinking, PCL10‐MA was melt‐mixed (80°C, 100 rpm, 30 min) with the radical photo‐initiator (2‐hydroxy‐2‐methylpropiophenone, 0.5 wt. %), then cooled to RT and kept in the dark until use.

In the following sections, T_XL_, t_XL_, and I_XL_ denote the crosslinking temperature, crosslinking time, and UV light intensity, respectively.

#### 2.3.1 2D Samples

**FIGURE 1:**
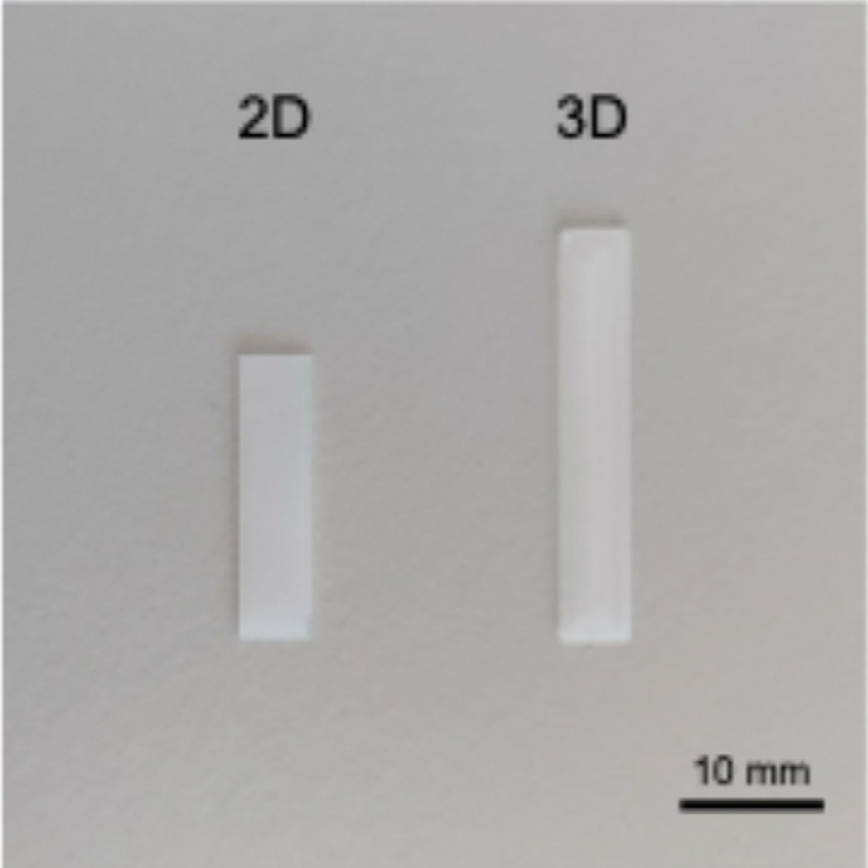
Representative 2D and 3D fabricated samples used for characterization.

The obtained mix was melted and poured between two glass slides (75 x 25 mm) and Teflon spacers (thickness: 0.4 mm) and gently compressed. Photo‐crosslinking was achieved by exposure to UV light (Hamamatsu LC8 spot light source) at λ = 365 nm for 120 s (i.e., t_XL_). Two crosslinking parameters were carefully controlled: the temperature (T_XL_ = 20°C and 60°C), to which the sample was equilibrated before UV exposure, and the UV light intensity (I_XL_ = 0.5 and 6 mW/cm^2^). Please note that the desired UV light intensity was set using a UV power meter (UV Light Checker C9386, Hamamatsu) with a glass slide interposed between the UV source and the meter, thereby accounting for the partial absorption of UV light by the glass. Free films with a thicknesses of about 0.4 mm were obtained by peeling them off from the glass slides. Rectangular specimens (20 x 5 x 0.4 mm, Figure 1) were then cut out from the cured samples and used for the subsequent characterization. Hereafter, specimens obtained via compression molding will be referred to as 2D.

Some of the samples obtained at T_XL_ = 20°C and I_XL_ = 0.5 mW/cm^2^ underwent post‐treatment (PT) after the first crosslinking step by heating them to T_XL_ = 60°C and irradiating them with high UV light intensity (I_XL_ = 6 mW/cm^2^) for an additional 120 s. Table 1 provides details of the post‐treatment process.

**TABLE 1:** Scheme of the prepared samples. In the sample name, 2D or 3D indicates the fabrication technique (FT), I_XL_ the UV light intensity, and T_XL_ the crosslinking temperature. Accordingly, the label was expressed as follows: FT_I_XL__T_XL_.

Sample name	FT	I_XL_ (mW/cm^2^)	T_XL_ (°C)	t_XL_ (s)
2D_0.5_20	2D	0.5	20	120
2D_6_20		6	20	120
2D_0.5_60		0.5	60	120
2D_6_60		6	60	120
2D_PT^a^		0.5 + 6	20 + 60	120 + 120
3D_0.5_20	3D	0.5	20	120
3D_6_20		6	20	120

^a^ Post‐treatment (PT) was performed by heating the sample 2D_0.5_20 to T_XL_ = 60°C and irradiating it with high UV light intensity (I_XL_ = 6 mW/cm^2^) for additional 120 s.

#### 2.3.2 3D Samples

Computer‐aided design (CAD) models (30 x 5 x 0.41 mm) were drawn in Autodesk Fusion (v. 2601.1.29) and processed to obtain. stl files, then imported into DNA Studio software (v. 4) for printing. Printing was carried out following a previously established protocol [29]. A pneumatic bioprinter (Cellink BioX6) equipped with a thermoplastic printhead and 22 G (diameter = 0.41 mm) nozzle size was used. The prepared mix was loaded into the print cartridge and heated to 70°C. Printing was achieved at a pressure of 15 kPa, speed of 10 mm/s, concentric infill (98% density), setting the print bed temperature at 20°C (i.e., T_XL_ = 20°C), and photo‐crosslinking was achieved using the integrated UV module (λ = 365 nm) of the 3D printer, irradiating the printed layer for 120 s (i.e., t_XL_) at different UV intensities (I_XL_ = 0.5 and 6 mW/cm^2^) after extrusion. The fabricated samples, consisting of single‐layer structures with dimensions of 30 x 5 x 0.41 mm (Figure 1) were used for the subsequent characterization. Hereafter, specimens obtained via 3D printing will be referred to as 3D.

For clarity, Table 1 provides a summary of all the prepared samples.

### 2.4 Characterization

#### 2.4.1 Gel Fraction

The gel fraction evaluation was carried out by weighting the samples after fabrication (w_0_), then placing them in THF at RT for 24 h using an immersion ratio of 0.5 g PCL to 15 mL THF. Following immersion, the swollen specimens were removed from the solvent and dried at RT for 24 h until a constant weight (w_d_) was reached, representing the residual weight after extraction. The gel content (G_f_ (%)) was calculated using the following equation (Equation (1)) [29]:

(1) $$G_{f}\;\left(\%\right)=\frac{w_{d}}{w_{0}}\times 100$$

#### 2.4.2 Thermal Characterization

The thermal properties of the samples were examined using Differential Scanning Calorimetry (DSC 250, TA Instruments), conducted over a temperature range of −20°C to 100°C at a heating/cooling rate of 10°C/min (heating/cooling/heating scans were carried out). The melting temperature (T_m_) and crystallization temperature (T_c_) were identified at the peak maximum of the endothermic transition and at the peak minimum of the exothermic transition, respectively. The degree of crystallinity ($\chi_{c}\left(\%\right)$) was calculated using the following equation (Equation (2)) [29]:

(2) $$\chi_{c}\left(\%\right)=\frac{\Delta H_{m}}{\Delta H_{m}^{100}}\;\times 100$$

where $\Delta H_{m}$ represents the melting enthalpy of the specimen in the second heating scan, $\Delta H_{m}^{100}$ was the theoretical melting enthalpy for 100% crystalline PCL (139.5 J/g [32]).

#### 2.4.3 Mechanical Characterization

The mechanical behavior of the samples was evaluated using a Dynamic Mechanical Analyzer (DMA Q850, TA Instruments) configured for tensile testing [29]. Quasi‐static tensile tests were conducted at 80°C, i.e., above the melting temperature of PCL (T > T_m_). The preload was set to 0.001 N, then a load ramp of 1 N/min was applied up to 18 N.

The Young's modulus (E) was determined from the slope of the stress‐strain curves in the initial linear range (ε = 0‐5%, R^2^ > 0.9) and the crosslinking density (ν) was calculated by applying the statistical Gaussian rubber theory following Equation (3) [16, 29]:

(3) E=3νRT

where E was the Young's modulus, R was the universal gas constant, and T was the absolute temperature.

#### 2.4.4 Shape‐Memory Characterization

The shape‐memory behavior of the samples was examined by evaluating the shape‐memory effect (SME), including both one‐way (1W) response and two‐way (2W) response, the latter assessed under either stress‐driven or stress‐free conditions [12, 29].

##### 2.4.4.1 One‐Way Shape‐Memory Effect

Before testing, the samples were first preconditioned at T = 80°C (i.e., above the melting temperature of PCL) for 10 min. The preload was set to 0.001 N, then a load ramp of 1 N/min was applied keeping the T = 80°C until a nominal strain ε_appl_ = 20% was achieved. Subsequently, keeping the strain constant (ε_appl_ = 20%), the samples were cooled down to −20°C (T < T_c_) at 2°C/min. Once the target temperature was reached, the load was removed (F = 0.001 N), and the specimens were heated up to 80°C at a constant heating rate of 2°C/min under quasi‐stress‐free conditions.

The material's ability to retain a temporary shape was quantified by calculating the strain fixity ratio ($R_{f}$), as defined in the following equation (Equation (4)):

(4) $$R_{f}\left(\%\right)=\frac{\varepsilon_{unload}}{\varepsilon_{appl}}\times 100$$

where $\varepsilon_{appl}$ was the applied nominal strain and $\varepsilon_{unload}$ was the strain after load removal.

The material's ability to recover its original (permanent) shape following the quasi‐stress‐free heating step was assessed by calculating the strain recovery ratio ($R_{r}$) according to the following equation (Equation (5)):

(5) $$R_{r}\left(\%\right)=\frac{\varepsilon_{appl}-\varepsilon_{rec}}{\varepsilon_{appl}}\times 100$$

where $\varepsilon_{appl}$ was the applied nominal strain and $\varepsilon_{rec}$ was the residual strain measured after the heating ramp.

##### 2.4.4.2 Stress‐Driven Two‐Way Shape‐Memory Effect

Before testing, the samples were first preconditioned at T = 80°C (i.e., above the melting temperature of PCL) for 10 min. The preload was set to 0.001 N, then a load ramp of 1 N/min was applied keeping the T = 80°C until a nominal strain ε_appl_ = 30% was achieved. (A higher strain value, ε_appl_ = 30%, was applied in the test to generate higher stresses in the specimens, which was necessary to observe a significant actuation magnitude). Then, keeping the force fixed (corresponding to ε_appl_ = 30%), a cooling‐heating cycle between −20 and 80°C (2°C/min rate) was carried out.

To quantitatively characterize the two‐way shape‐memory behavior, three parameters were determined: the actuation magnitude (AM), the recovery magnitude (RM), and the the stress‐driven reversible deformation ($\Delta \varepsilon_{rev(stress-driven)}$) as defined in Equations (6)–(8) respectively.

(6) $$AM\;\left(\%\right)=\varepsilon_{low}-\varepsilon_{appl}$$

(7) $$RM\;\left(\%\right)=\frac{\varepsilon_{low}-\varepsilon_{high}}{\varepsilon_{low}-\varepsilon_{appl}}\times 100$$

(8) $$\Delta \varepsilon_{rev\left(stress-driven\right)}\;\left(\%\right)=\varepsilon_{low}-\varepsilon_{high}$$

where $\varepsilon_{appl}$ represents the strain applied at 80°C (30% nominal), $\varepsilon_{low}$ the strain after cooling under load at −20°C, and $\varepsilon_{high}$ the recovered strain at the end of the following heating under load at 80°C.

##### 2.4.4.3 Stress‐Free Two‐Way Shape‐Memory Effect

Before testing, the samples were first preconditioned at T = 80°C (i.e., above the melting temperature of PCL) for 10 min. The preload was set to 0.001 N, then a load ramp of 1 N/min was applied keeping the T = 80°C until a nominal strain ε_appl_ = 30% was achieved. (A higher strain value (ε_appl_ = 30 vs. 20%) was applied in the test to generate higher stresses in the specimens, which was necessary to observe a significant reversible actuation.) Then, keeping the force fixed (corresponding to ε_appl_ = 30%), a cooling ramp at 2°C/min was applied down to −20°C. Subsequently, the sample was unloaded (F = 0.001 N) and heated under quasi‐stress‐free conditions (2°C/min) up to a selected actuation temperature (T_act_). Then the sample was cooled to −20°C (2°C/min), and a second heating/cooling cycle, achieving a second T_act_, was performed before the final heating to 80°C.

Please, note that preliminary tests were carried out to select the actuation temperatures (data not shown). These tests followed the same procedure as the stress‐free two‐way tests, except for the final heating step. Instead of heating up to the T_act_, the specimens were heated up to 80°C, undergoing full recovery. From each strain‐temperature curve, in the recovery step, two T_act_ were selected after the beginning of the recovery of ε.

To quantitatively characterize the stress‐free two‐way shape‐memory behavior, the stress‐free reversible deformation ($\Delta \varepsilon_{rev}$) was calculated as defined in Equation (9):

(9) $$\Delta \varepsilon_{rev\left(stress-free\right)}\;\left(\%\right)=\varepsilon_{max}-\varepsilon_{min}$$

where $\varepsilon_{max}$ and $\varepsilon_{min}$ represent the maximum and minimum strains achieved during the reversible cooling/heating cycle.

## 3 Results and Discussion

The aim of this work was to explore how selected crosslinking parameters and different fabrication techniques influence the thermo‐mechanical, physical, and shape‐memory properties of semi‐crystalline PCL‐based networks. In details, the explored crosslinking parameters were: i) the crosslinking temperature (T_XL_ = 20 or 60°C), below the crystallization temperature (T_c_) or above the melting temperature (T_m_) of PCL, to explore the effect of the material's state (in the presence or absence of a crystalline phase) during crosslinking. ii) The UV light intensity (I_XL_ = 0.5 or 6 mW/cm^2^), the two values being selected as corresponding to the lowest and highest intensity achievable in the operational range of the printing machine (Cellink BioX6).

Crosslinking times were kept constant (t_XL_ = 120 s) based on a previous study [29]. Specimens fabricated via 2D or 3D technique were then compared, to investigate the effect of the printing process on the behavior of the obtained samples.

Please note that a T_XL_ = 60°C was not investigated with 3D printed specimens, as shape fidelity could not be maintained at this temperature. For further details, the reader is referred to [29].

### 3.1 Physical and Thermal Properties of the Samples

**FIGURE 2:**
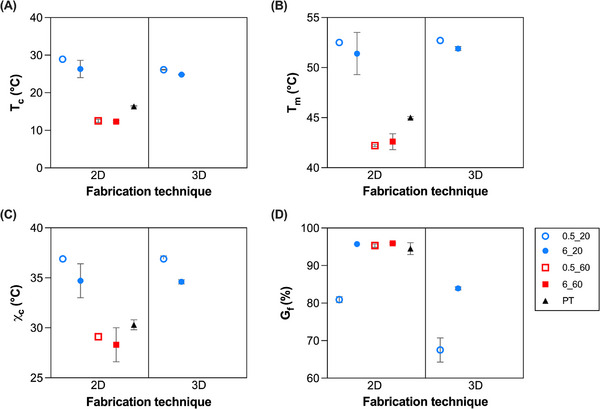
Thermal and physical properties of the semi‐crystalline PCL networks prepared: (A) crystallization temperature, T_c_; (B) melting temperature, T_m_; (C) degree of crystallinity, χ_c_; (D) gel fraction, G_f_. Blue dots: T_XL_ = 20°C, red squares: T_XL_ = 60°C. Empty symbol: I_XL_ = 0.5 mW/cm^2^, full symbol: I_XL_ = 6 mW/cm^2^. Black triangle = PT. (For interpretation of the references to color in this figure legend, the reader is referred to the web version of this article.).

The obtained specimens were characterized in terms of gel fraction by immersion in THF. Thermal parameters were determined through DSC analysis. The resulting data are presented in Table 2 and Figure 2.

**TABLE 2:** Thermal and physical properties of semi‐crystalline PCL networks prepared.

Sample name	T_c_ (°C)	T_m_ (°C)	ΔH_m_ (J/g)	χ_c_ (%)	G_f_(%)
2D_0.5_20	28.9 ± 0.3	52.5 ± 0.0	51.5 ± 0.0	36.9 ± 0.0	80.9 ± 0.8
2D_6_20	26.3 ± 2.3	51.4 ± 2.1	48.4 ± 2.4	34.7 ± 1.7	95.7 ± 0.5
2D_0.5_60	12.5 ± 0.4	42.2 ± 0.1	40.5 ± 0.0	29.1 ± 0.0	95.3 ± 0.4
2D_6_60	12.3 ± 0.3	42.6 ± 0.8	39.5 ± 2.4	28.3 ± 1.7	95.9 ± 0.2
2D_PT	16.4 ± 0.2	45.0 ± 0.1	42.2 ± 0.7	30.3 ± 0.5	94.5 ± 1.6
3D_0.5_20	26.1 ± 0.1	52.7 ± 0.2	51.4 ± 0.4	36.9 ± 0.3	67.5 ± 3.2
3D_6_20	24.8 ± 0.1	51.9 ± 0.2	48.2 ± 0.2	34.6 ± 0.2	83.9 ± 0.4

As it is possible to observe by comparing the 2D specimens, the crosslinking parameters, i.e., T_XL_ and I_XL_, strongly influenced the thermal and physical properties of the obtained samples. Crosslinking at T_XL_ = 20°C led to high values of T_m_, T_c_, and crystallinity content (χ_c_). Instead, crosslinking at T_XL_ = 60°C, when the sample is fully melted, led to a decrease in the T_m_ and T_c_ of the crosslinked specimen, as well as in their χ_c_. In particular, the T_m_ was reduced of about 10°C (Figure 2B), while the T_c_ of about 15°C (Figure 2A). Accordingly, a decrease in χ_c_ of about 8% was observed (Figure 2C) in the samples crosslinked at T_XL_ = 60°C.

The decrease of the degree of crystallinity can be ascribed to a hindered crystallization process, due to the restricted chain mobility in the presence of a larger amount of chemical net points. Moreover, the physical state of the sample ‐in the presence or absence of a crystalline phase‐ possibly influences the spatial distribution of crosslinking points within the material. The associated decrease in the melting/crystallization temperatures can be ascribed to stricter bonds between chains, resulting in a less perfected structure of the crystals [29, 33].

In terms of gel fraction, most of the specimens displayed high gel fraction values of ≈95% (Figure 2D). Such results are in line with previous works, where G_f_ values in the 90–94% range were found for UV‐crosslinked PCL10‐PEG semi‐crystalline networks [12] and PCL10 crosslinked by sol–gel chemistry [16]. Conversely, sample 2D_0.5_20 displayed a lower G_f_ compared with other specimens (≈80 vs. ≈95%) due to the lower crosslinking extent achieved in this specimen. The results thus suggest that by either increasing I_XL_ or increasing T_XL_ above melting temperature promotes an increase in the crosslinking efficiency.

Interestingly, the impact of the post‐treatment (PT) on the thermal properties of the specimens was found to be noteworthy. Specifically, this treatment led to an important decrease of T_m_, T_c_, and χ_c_ values, that reached values closer to those of the specimens treated at T_XL_ = 60°C.

The fabrication technique (i.e., 2D vs. 3D) did not appear to have an impact on the thermal properties of the samples, but rather to influence the physical ones. In particular, the gel fraction values of the 3D printed specimens resulted significantly lower than those of 2D ones (Figure 2D). Similar observations were made in our previous work [29], where lower gel fraction values (≈85%) were obtained for 3D printed specimens, in line with the G_f_ of sample 3D_6_20. These outcomes can be ascribed to differences in the degree of crosslinking achieved during 3D printing, most likely arising from the inhibitory effect of oxygen on crosslinking. Oxygen present in the air has a well‐documented inhibitory effect on the photopolymerization of acrylate and methacrylate groups by forming a superficial inhibited layer, usually referred to as the OIL (oxygen inhibition layer). At the surface exposed to air, oxygen diffuses easily and reacts with the radicals generated by light, forming less reactive peroxide radicals. This “consumes” the active radicals, slowing down or blocking the polymerization reaction [34]. Notably, this effect is more pronounced in 3D specimens, as 2D specimens are processed between two glass slides, which markedly limits OIL formation.

### 3.2 Mechanical Properties of the Samples

**FIGURE 3:**
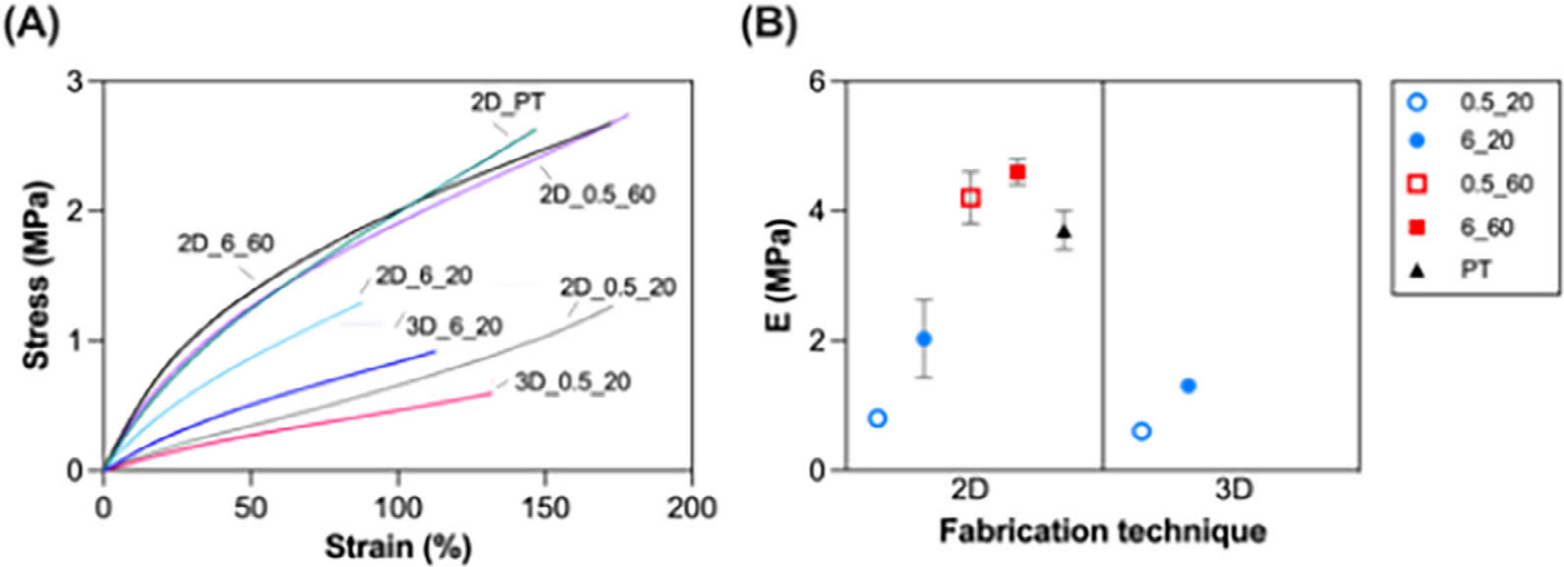
Mechanical characterization of semi‐crystalline PCL networks prepared. (A) Representative σ/ε curves and (B) Young's moduli obtained from QS tensile tests at T = 80°C. Blue dots: T_XL_ = 20°C, red squares: T_XL_ = 60°C. Empty symbol: I_XL_ = 0.5 mW/cm^2^, full symbol: I_XL_ = 6 mW/cm^2^. Black triangle = PT. (For interpretation of the references to color in this figure legend, the reader is referred to the web version of this article.).

Quasi‐static tensile tests were carried out on the samples at 80°C (T > T_m_). Data obtained from these analyses are reported in Tables 2, 3, and Figure 3. It is worth clarifying that the specimens did not break within the test window, but all reached the displacement limit of the machine (depending on their initial length).

**TABLE 3:** Mechanical properties derived from QS tensile tests on the semi‐crystalline polymer networks prepared.

Sample name	E (MPa)	Crosslinking density, ν (10^−4^ mol cm^−3^)
2D_0.5_20	0.8 ± 0.1	0.9 ± 0.1
2D_6_20	2.0 ± 0.6	2.3 ± 0.7
2D_0.5_60	4.2 ± 0.4	4.8 ± 0.4
2D_6_60	4.6 ± 0.2	5.3 ± 0.2
2D_PT	3.7 ± 0.3	4.2 ± 0.3
3D_0.5_20	0.6 ± 0.0	0.7 ± 0.0
3D_6_20	1.3 ± 0.0	1.5 ± 0.0

As expected, stress‐strain curves (Figure 3A) were highly influenced by the crosslinking parameters. Considering 2D specimens, an increase in the Young's modulus (Figure 3B) was observed by increasing the UV power intensity, particularly for specimens crosslinked at low temperatures (E = 0.8 ± 0.1 vs. 2.0 ± 0.6 MPa for 2D_0.5_20 and 2D_6_20, respectively). The influence of the crosslinking temperature was even more noticeable, leading to a 5‐fold increase in the E values comparing specimens irradiated at the same UV power intensity (E = 0.8 ± 0.1 vs. 4.2 ± 0.4 MPa for 2D_0.5_20 and 2D_0.5_60, respectively).

The E values obtained in this work are consistent with our previous findings on PCL10 semi‐crystalline networks photo‐crosslinked via UV light. Specifically, previous samples crosslinked at T_XL_ = 60°C and I = 6 mW/cm^2^ exhibited E = 4.55 ± 0.16 MPa [35], matching that of the 2D_6_60 samples (E = 4.6 ± 0.2 MPa, Table 3). The same observations done for the Young's modulus can be made for the crosslinking density (ν, Table 3).

Also in terms of mechanical performance, the impact of the post‐treatment on the specimens was noteworthy. Specifically, this treatment resulted in a significant increase in the E values (E = 3.7 ± 0.3 for 2D_PT), approaching those observed under high crosslinking conditions. Accordingly, the crosslinking density increased, likely because the post‐treatment targeted domains that were crystalline at T < T_c_ and therefore inaccessible to crosslinking [36]. Upon melting (T > T_m_), these regions became amorphous and accessible to additional crosslinking.

Interestingly, the fabrication technique was also disclosed here to influence the mechanical properties of the specimens, especially for samples crosslinked at high UV power intensity (6 mW/cm^2^). In fact, by comparing 2D_6_20 and 3D_6_20 specimens, a slight decrease in the Young's modulus was observed (E = 2.0 ± 0.6 vs. 1.3 ± 0.0 MPa for 2D_6_20 and 3D_6_20, respectively). Such observations can be related to inter‐filament bonding issues. This latter issue has been highlighted in numerous studies unveiling how issues in mechanical continuity, particularly related to inter‐filament and inter‐layer bonding, are intrinsic to extrusion‐based 3D printing techniques. These issues stem mainly from the nature of the extrusion‐based printing process, which can result in structural defects and anisotropic mechanical properties in printed parts [37, 38].

### 3.3 Shape‐Memory Properties of the Samples

#### 3.3.1 One‐Way SME

**FIGURE 4:**
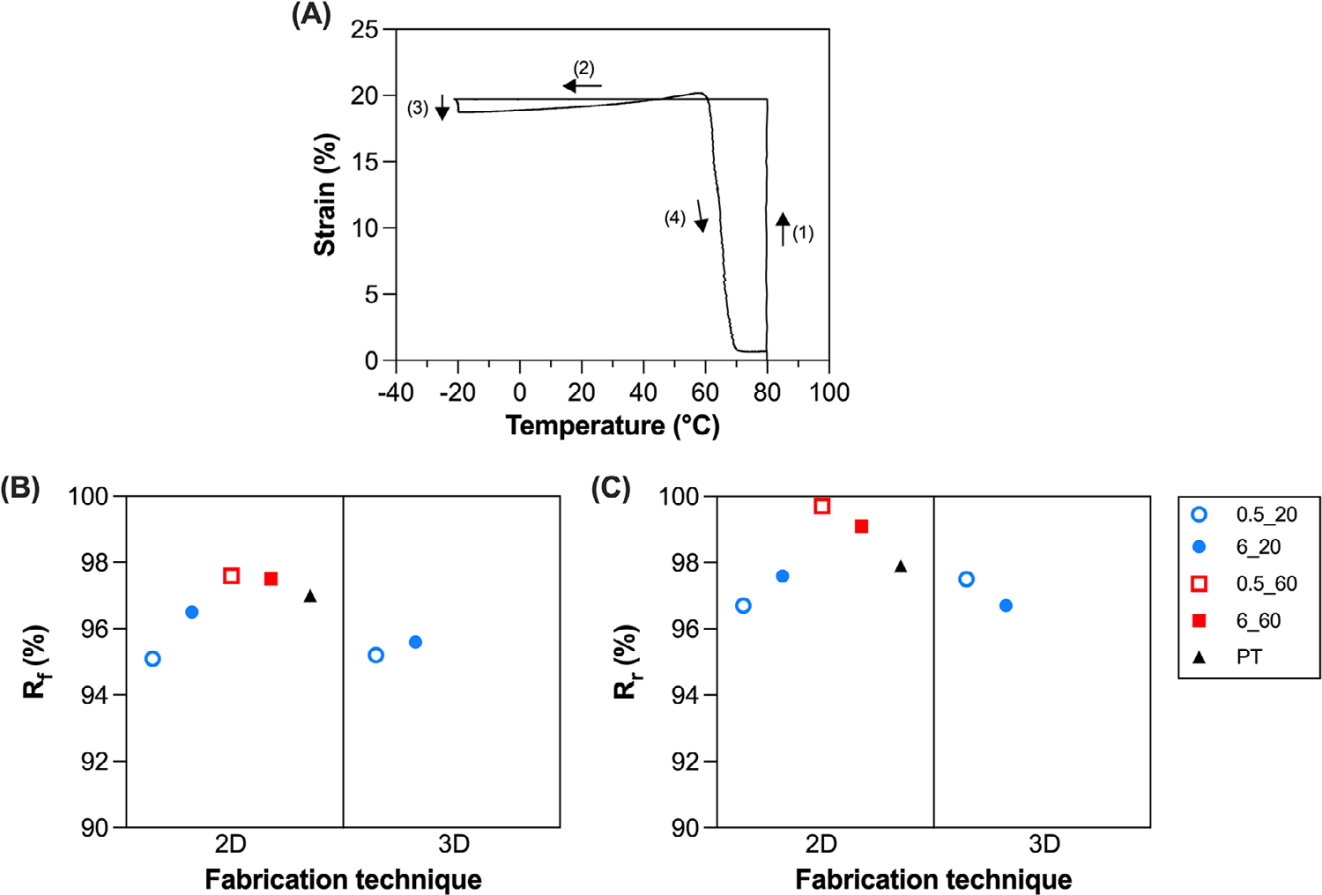
One‐way shape‐memory effect of the semi‐crystalline PCL networks prepared. (A) Representative one‐way shape‐memory test: (1) the specimen is stretched to ε_appl_ = 20% at 80°C, (2) cooled to −20°C while maintaining constant strain (ε_appl_ = 20%), (3) unloaded (F = 0.001 N), and finally (4) reheated to 80°C under quasi stress‐free conditions (F = 0.001 N). (B) Strain fixity ratio, R_f_, and (C) strain recovery ratio, R_r_, obtained from one‐way shape‐memory tests. Blue dots: T_XL_ = 20°C, red squares: T_XL_ = 60°C. Empty symbol: I_XL_ = 0.5 mW/cm^2^, full symbol: I_XL_ = 6 mW/cm^2^. Black triangle = PT. (For interpretation of the references to color in this figure legend, the reader is referred to the web version of this article.).

The one‐way shape‐memory behavior of the samples was evaluated using a defined thermo‐mechanical cycle via DMA (Figure 4A).

The high R_f_ values (R_f_ ≥ 95%, Figure 4B, Table 4) obtained for all the tested specimens demonstrate that the temporary shape was effectively retained after unloading, indicating efficient crystallization during cooling. Similarly, high R_r_ value (R_r_ ≥ 97%, Figure 4C, Table 4) confirmed nearly complete recovery of the permanent shape upon reheating above T_m_ [39].

**TABLE 4:** One‐way shape‐memory properties of the semi‐crystalline PCL networks prepared.

Sample name	ε_appl_ (%)^a^	R_f_ (%)	R_r_ (%)
2D_0.5_20	20	95.1	96.7
2D_6_20		96.5	97.6
2D_0.5_60		97.6	99.7
2D_6_60		97.5	99.1
2D_PT		97.0	97.9
3D_0.5_20	20	95.2	97.5
3D_6_20		95.6	96.7

^a^ = nominal strain.

This analysis indicates that, although slightly, high T_XL_ and I_XL_ values positively influenced the one‐way shape‐memory performance. In particular, a high T_XL_ led to a slight (≈ 1–2%) increase in the R_f_ (96.5 vs. 97.5% for 2D_6_20 and 2D_6_60, respectively) and R_r_ (97.6 vs. 99.1% for 2D_6_20 and 2D_6_60, respectively) compared to a low T_XL_.

Interestingly, the fabrication technique was not found to have a significant influence on the one‐way shape‐memory behavior of the specimens. These results align well with our previous studies on PCL‐based SMPs fabricated both through traditional fabrication techniques [16, 39] and additive manufacturing [29], both displaying R_f_ and R_r_ values ≥ 95%. Therefore, it can be concluded that the printing process does not impair the material's excellent one‐way shape‐memory performance.

#### 3.3.2 Stress‐Driven Two‐Way SME

The two‐way SME was then assessed to evaluate the potential of the obtained semi‐crystalline PCL networks for reversible shape transformation. First, reversibility was assessed under mechanical load (i.e., stress‐driven conditions) by applying a thermal cooling/heating cycle. Similar to the one‐way SME, the two‐way SME results from the interplay between the macromolecular architecture of the polymer network and its thermo‐mechanical history [3, 29].

In particular, to achieve two‐way SME in semi‐crystalline polymer networks, both a crystallizable phase and physical or chemical net points must be present. Under a constant tensile load, the polymer network elongates upon cooling due to two distinct mechanisms, namely the entropy elasticity effect in the rubbery region and the crystallization‐induced effect close to T_c_ [5]. Conversely, heating the material above its T_m_ causes contraction, which is attributed to the melting of previously oriented crystalline domains. Therefore, it can be concluded that the two‐way SME is based on a crystallization‐induced elongation (CIE) process during cooling and on a melting‐induced contraction (MIC) process during heating [3, 5, 40].

**FIGURE 5:**
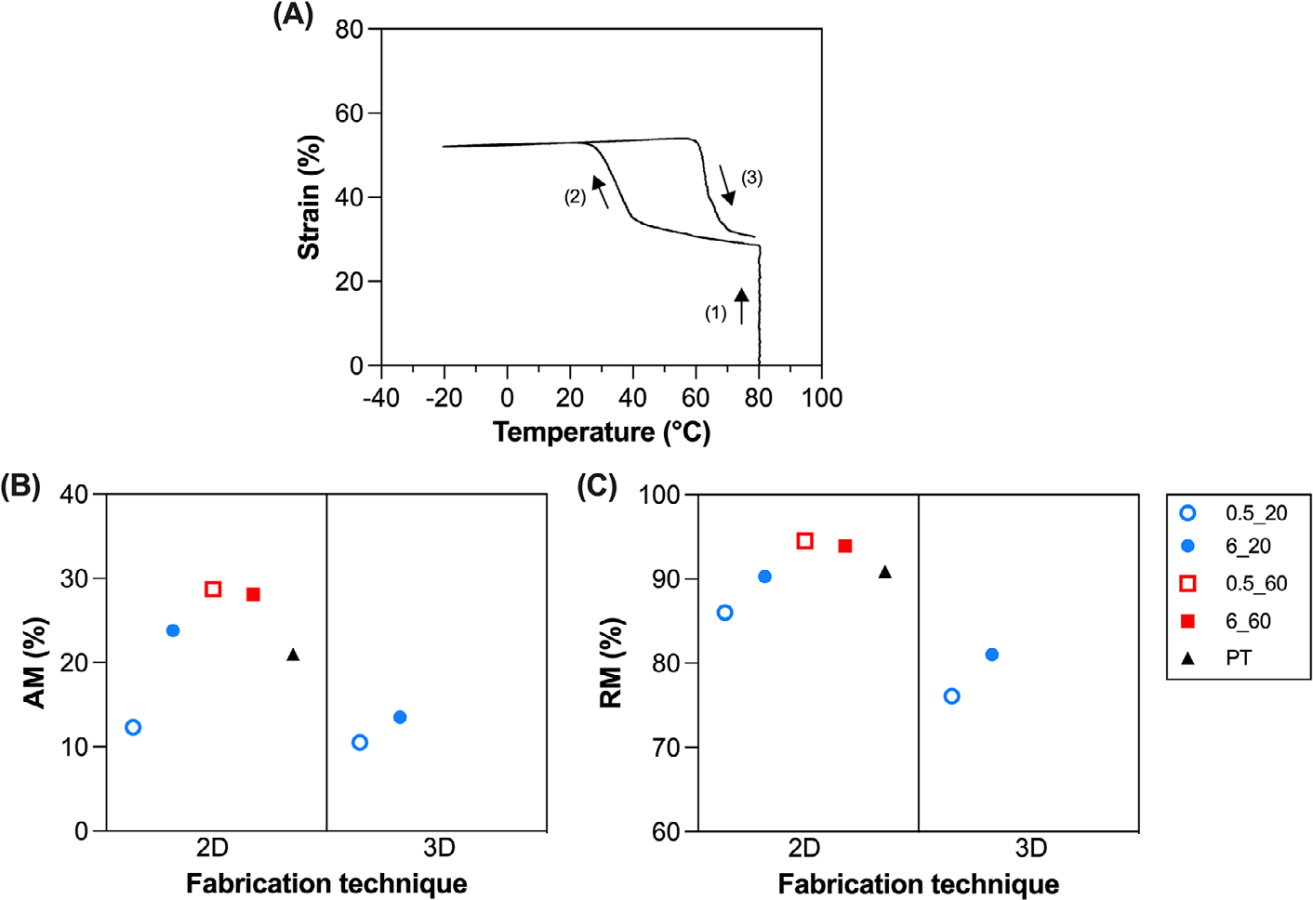
Two‐way shape‐memory effect under an applied load (i.e., stress‐driven) of the semi‐crystalline PCL networks prepared. (A) Representative two‐way shape‐memory test under load: (1) a controlled load ramp of 1 N/min is applied at 80°C (i.e., T > T_m_) until a strain level of 30% is reached. Maintaining the force constant, corresponding to the strain obtained at the end of this step, a (2) cooling and (3) heating cycle between −20°C and 80°C is then executed at a controlled rate of 2°C/min. (B) Actuation magnitude, AM, and (C) recovery magnitude, RM, obtained from two‐way shape‐memory tests under load. Blue dots: T_XL_ = 20°C, red squares: T_XL_ = 60°C. Empty symbol: I_XL_ = 0.5 mW/cm^2^, full symbol: I_XL_ = 6 mW/cm^2^. Black triangle = PT. (For interpretation of the references to color in this figure legend, the reader is referred to the web version of this article).

The stress‐driven two‐way SME of the samples was evaluated using a defined thermo‐mechanical cycle via DMA (Figure 5A). This thermo‐mechanical cycle allowed the evaluation of the reversible strain changes resulting from CIE during cooling and MIC during heating (Table 5).

**TABLE 5:** Two‐way shape‐memory properties under an applied load (i.e., stress‐driven) of the semi‐crystalline PCL networks prepared.

Sample name	ε_appl_ (%)^a^	σ (kPa)	AM (%)	$\Delta \varepsilon_{rev(stress-driven)}$ (%)	RM (%)	T_CIE_ (°C)^b^	T_MIC_ (°C)^b^
2D_0.5_20	30	284.4	12.3	10.5	86.0	44.6	65.6
2D_6_20		833.2	23.8	21.5	90.3	40.0	61.1
2D_0.5_60		1075.5	28.7	27.5	94.5	27.9	56.9
2D_6_60		1056.9	28.1	26.4	93.9	26.0	59.2
2D_PT		924.8	21.0	19.1	90.9	32.3	60.2
3D_0.5_20	30	246.2	10.5	8.0	76.1	43.6	59.1
3D_6_20		361.2	13.5	11.0	81.0	40.5	57.7

^a^ nominal strain;

^b^ T_CIE_ was calculated from the onset point of the steepest elongation process in the ε vs. T curve, while T_MIC_ was calculated from the onset point of the steepest contraction process in the ε vs. T curve.

The actuation magnitude (AM) and recovery magnitude (RM) were calculated using Equations (6) and (7) (Figure 5 and Table 5).

The AM values (Figure 5B) were strongly influenced by the crosslinking parameters, with the crosslinking temperatures playing the most critical role. Indeed, when the UV intensity was kept constant (I_XL_ = 0.5 mW/cm^2^), more than a 2‐fold increase in the AM values was obtained at the two crosslinking temperatures (AM = 28.7 and 12.3% for 2D_0.5_60 and 2D_0.5_20, respectively). Such an effect was less noticeable at higher UV light intensity (AM = 28.1 and 23.8% for 2D_6_60 and 2D_6_20, respectively), possibly because, at high temperature, the crosslinking plateau is already reached, whereas, at low temperature, a higher UV intensity is required to achieve the same level of crosslinking. Consistent with this observation, the effect of the UV intensity on the extent of the AM was more noticeable at low T_XL_, where almost a 2‐fold increase in the AM was obtained at the two UV light intensities explored (AM = 23.8 and 12.3% for 2D_6_20 and 2D_0.5_20, respectively).

It is important to evidence a correlation between the AM and the applied stress. Since the tested samples exhibited different crosslinking degrees, different values of stress were required to reach the target strain of 30% (Table 5). It is well known in the literature that a higher applied stress generally results in increased AM [22], a trend that is also reflected in the data presented in this work. However, this is likely not the only factor influencing the AM. In fact, when comparing samples 2D_6_60 and 2D_PT, which experienced similar stress levels, marked differences in AM were observed. This difference may be attributed to variations in the three‐dimensional network architecture. Specifically, the different crosslinking conditions likely influenced the spatial distribution of the crosslinking points within the polymer network, ultimately impacting on the AM values.

In terms of RM values (Figure 5C), less pronounced differences were observed among the 2D specimens, with RM values moving from 86% for less crosslinked samples (2D_0.5_20) to 93.9% for the more crosslinked ones (2D_6_60).

These AM and RM values obtained for high crosslinking conditions (AM = 28.1%, RM = 93.9% for 2D_6_60) are consistent with earlier findings on PCL‐based shape‐memory polymers obtained by both sol‐gel crosslinking (AM = 26%, RM = 94%, ε_appl_ = 40%) [16] and UV crosslinking (AM = 25%, RM = 97%, ε_appl_ = 20%) [35].

The post‐treatment resulted in an overall enhancement of the two‐way shape‐memory performance of the specimens. Notably, it had a positive impact on the AM values, that reached values (AM = 21.0% for 2D_PT) midway between the low (AM = 12.3% for 2D_0.5_20) and high (AM = 28.1% for 2D_6_60) crosslinking conditions. As already stated, these observations can be related to a combination of factors: i) the higher crosslinking degree achieved through the post‐treatment process, which leads to increased applied stress (at 30% strain), and ii) a different spatial distribution of the crosslinking points.

The fabrication technique was also disclosed here to influence the two‐way shape‐memory performance of the specimens, especially for samples crosslinked at high I_XL_. In fact, by confronting 2D_6_20 and 3D_6_20 specimens, a 1.8‐fold decrease in the AM (AM = 23.8 and 13.5% for 2D_6_20 and 3D_6_20, respectively) and a 1.1‐fold decrease in the RM (RM = 90.3 and 81% for 2D_6_20 and 3D_6_20, respectively) were observed. As previously mentioned, these observations can be related to both a reduced crosslinking (resulting in lower stress required to reach 30% strain) most likely arising from the inhibitory effect of oxygen and inter‐filament bonding issues. Such results indicate that 3D printing can negatively affect the performance of the specimens, especially when a reversibility of the shape change is needed. Possible strategies to mitigate these drawbacks will be envisaged in Section 3.4.

#### 3.3.3 Stress‐Free Two‐Way SME

**FIGURE 6:**
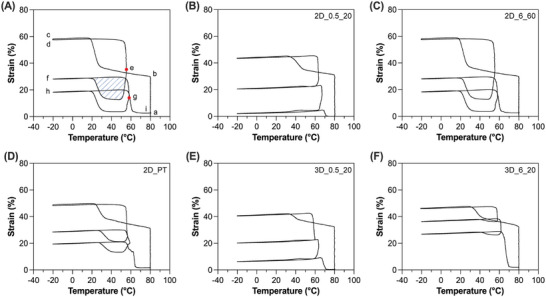
Two‐way shape‐memory test without load applied (i.e., stress‐free) of the semi‐crystalline PCL networks prepared. (A) Representative strain vs. temperature plot: (a→b) a controlled load ramp of 1 N/min is applied at 80°C (i.e., T > T_m_) until a strain level of 30% is reached. Maintaining the force constant, corresponding to the strain obtained at the end of this step, (b→c) the specimen is cooled down to −20°C (i.e., T < T_c_). Then, (c→d) the load is removed (F = 0.001 N) and (d→e) the material is heated at a given so‐called actuation temperature (T_act_, corresponding to point e) to promote partial melting of the crystalline phase and associated partial recovery; next, (e→f) the sample is cooled to −20°C and (f→g)/(g→h) a second heating/cooling cycle, achieving a second T_act_ (corresponding to point g) is performed, before (h→i) the final heating to 80°C. The red dots represent the T_act_, while the dotted blue area represents the elongation‐contraction cycle. (B) 2D_0.5_20, (C) 2D_6_60, (D) 2D_PT, (E) 3D_0.5_20, (F) 3D_6_20. (For interpretation of the references to color in this figure legend, the reader is referred to the web version of this article.).

Reversible shape‐memory effect was then assessed in the absence of mechanical load (i.e., stress‐free conditions) on selected specimens (Figure 6, Table 6). Such an effect can be explored in semi‐crystalline homopolymer networks by exploiting part of the crystals (cyclically crystallizing and melting) as the actuator domain, and the remaining part of the crystals as the so‐called skeleton domain [11, 41, 42].

**TABLE 6:** Two‐way shape‐memory effect without load applied (i.e., stress‐free) of the semi‐crystalline PCL networks prepared.

Sample name	ε_app_ (%)^a^	σ (kPa)	T_act_ (°C)	$\Delta \varepsilon_{rev(stress-free)}$ (%)	T_CIE_ (°C)^b^
2D_0.5_20	30	279.0	63.7	—	—
			67.3	—	—
2D_6_60		987.1	55.5	16.9	30.2
			58.4	16.4	28.8
2D_PT		906.2	56.7	8.9	38.2
			59.2	7.7	38.1
3D_0.5_20	30	217.7	59.9	—	—
			63.8	—	—
3D_6_20		361.5	58.0	2.1	48.5
			61.7	2.7	49.8

^a^ nominal strain;

^b^ T_CIE_ was calculated from the onset point of the steepest elongation process in the ε vs. T curve.

The stress‐free two‐way SME of the samples was evaluated using a defined thermo‐mechanical cycle via DMA (Figure 6A).

The response is an elongation‐contraction cycle (represented by the blue dotted area, Figure 6A) under no applied load, that may be considered as a reversible two‐way effect. Such reversible behavior during each cooling‐heating cycle (between T_act_ and −20°C) may be considered as a result of the thermo‐mechanical history, acting as a sort of training of the material for the self‐standing response [11].

It is interesting to notice that crosslinking at low temperature and low UV intensity (T_XL_ = 20°C, I_XL_ = 0.5 mW/cm^2^, Figures 6B,E) resulted into no stress‐free reversible deformation ($\Delta \varepsilon_{rev(stress-free)}$). A higher UV intensity (6 mW/cm^2^) only led to a slight increase in the $\Delta \varepsilon_{rev(stress-free)}$ (Figure 6F). Conversely, crosslinking at high temperature and high UV intensity (T_XL_ = 60°C, I_XL_ = 6 mW/cm^2^, Figure 6C) resulted in a noteworthy $\Delta \varepsilon_{rev(stress-free)}$ of about 16%.

A possible explanation of this observation is that the stress‐free two‐way SME is strictly dependent on the three‐dimensional network architecture, that is clearly different according to the selected crosslinking temperature. In fact, looking at the physical and thermal properties of the samples (Table 2), higher crosslinking conditions (2D_6_60) led to a lower crystalline fraction compared to lower crosslinking temperatures (2D_6_20), with χ_c_ = 28.3 ± 1.7 vs. 36.9 ± 1.7%, respectively. Instead, the gel fraction values obtained were comparable and around 95–96%. The crosslinking density (Table 3) was also found to increase as a function of the crosslinking temperature, with values of (5.3 ± 0.2) ×10^−4^ and (2.3 ± 0.7) ×10^−4^ mol cm^−3^ for 2D_6_60 and 2D_6_20, respectively.

Overall, in line with the observations made for the stress‐driven two‐way SME, the stress applied to the specimens (Table 6) may influence, in the first instance, the magnitude of the $\Delta \varepsilon_{rev(stress-free)}$ observed in the two‐way stress‐free SME [11]. Specifically, since different stress values (corresponding to 30% strain) were applied due to the different degrees of crosslinking, the $\Delta \varepsilon_{rev(stress-free)}$ varied accordingly.

However, this explanation alone is unlikely to fully account for the observed behavior. Probably, differences in the spatial distribution of crosslinking points within the polymer network also play a significant role, ultimately affecting the $\Delta \varepsilon_{rev(stress-free)}$ values. (On this point, further details will be provided in Section 3.4.)

Notably, the post‐treatment resulted in an overall enhancement of the stress‐free two‐way SME of the specimens, positively impacting the $\Delta \varepsilon_{rev(stress-free)}$, that reached values ($\Delta \varepsilon_{rev(stress-free)}$ ≈ 8–9% for 2D_PT) midway between the low ($\Delta \varepsilon_{rev(stress-free)}$ = 0% for 2D_0.5_20) and high (AM ≈ 16% for 2D_6_60) crosslinking conditions.

The effect of the fabrication technique on the two‐way shape‐memory behavior under stress‐free conditions was not appreciable, primarily due to the lack of reversible actuation even in the 2D specimens.

It is interesting to notice that the T_act_ seems not to influence the $\Delta \varepsilon_{rev(stress-free)}$ values (Table 6). Instead, the history of the samples (e.g., crosslinking parameters) seems to play a more dominant role. These results are consistent with a previous study on PCL‐based semi‐crystalline polymer networks prepared via sol–gel chemistry [11], where low‐molecular‐weight (2–3 kDa) PCL networks exhibited an increase in $\Delta \varepsilon_{rev(stress-free)}$ with T_act_, whereas this effect was not observed in higher‐molecular‐weight (10 kDa) PCL.

It should also be noted that, although the objective of this study was not to investigate the repeatability of stress‐free actuation, reversibility is nevertheless expected when cyclic thermal ramps are applied between T_low_ (−20°C) and a fixed T_act_. Indeed, we previously observed this behavior in other semi‐crystalline polymer networks based on PCL [12], where three consecutive heating/cooling cycles consistently produced reversible strain evolution without any measurable loss of performance. For further details the reader is referred to [12].

### 3.4 Guidelines for the Design of Structures With Optimal Shape‐Memory Properties

As evidenced by the results obtained in this study, the crosslinking parameters and the fabrication technique are two critical factors that significantly influence material properties from a physico‐structural, mechanical, and shape‐memory standpoint.

**FIGURE 7:**
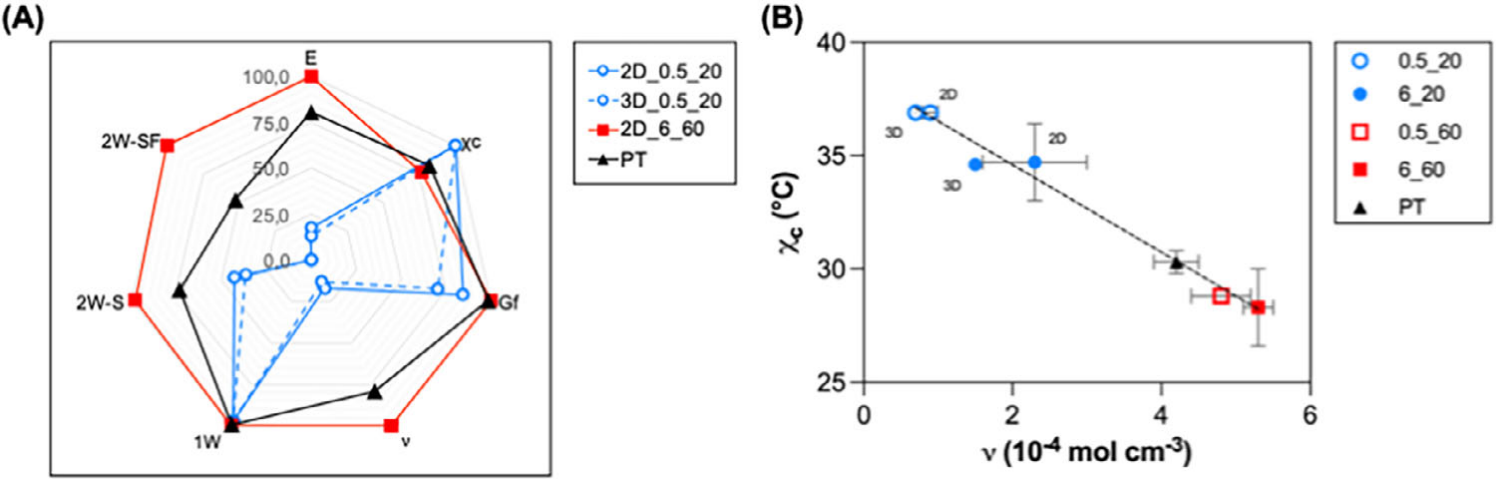
(A) Radar chart displaying the overall performance of the selected samples in terms of physical, mechanical, and shape‐memory behavior. Such a graph was obtained by normalizing the data with respect to the local maximum, i.e., setting the highest value of each parameter to 100. (B) χ_C_ vs. ν plot of semi‐crystalline PCL networks prepared. The dotted black line is the linear regression curve (R^2^ > 0.9) that aims to represent the general trend of the dataset. Blue dots: T_XL_ = 20°C, red squares: T_XL_ = 60°C. Empty symbol: I_XL_ = 0.5 mW/cm^2^, full symbol: I_XL_ = 6 mW/cm^2^. Black triangle = PT. (For interpretation of the references to color in this figure legend, the reader is referred to the web version of this article.).

Figure 7A presents the overall performance of the selected samples through a radar chart. Such a graph was obtained by normalizing the data with respect to the local maximum, i.e., setting the highest value of each parameter to 100. Please, note that 1W, 2W‐S, and 2W‐SF represent the recovery ratio (R_r_) in one‐way shape‐memory tests, the actuation magnitude (AM) in stress‐driven two‐way shape‐memory tests, and the stress‐free reversible deformation ($\Delta \varepsilon_{rev(stress-free)}$) in stress‐free two‐way shape‐memory tests, respectively.

The overall performance of the samples can be, in the first instance, evaluated through the area of each sample type on the radar chart. As already observed, a high crosslinking temperature (T_XL_ > T_m_) enables the obtainment of structures with superior mechanical performance that also display stress‐free reversible shape‐memory properties. Conversely, a low crosslinking temperature (T_XL_ < T_c_) results in reduced mechanical performance and absence of stress‐free reversible shape‐memory behavior.

This behavior is likely attributable to the macromolecular architecture of the crosslinked networks. Specifically, at T_XL_ = 20°C, the presence of crystalline domains (and the resulting limited chain mobility) constrains the crosslinking process, which predominantly occurs in the amorphous regions of the PCL chains [36]. This results in a three‐dimensional network with a presumably irregular distribution of crosslinking points throughout the volume, leading to a lower overall crosslinking density.

In contrast, at T_XL_ = 60°C, the increased chain mobility and absence of crystalline hindrance likely promote a more regular spatial distribution of crosslinking points throughout the volume, resulting in a higher crosslinking density and a tighter network structure. Interestingly, noteworthy differences in the ν values (Table 3, Figure 7A) were observed even when similar values of G_f_ were obtained (e.g., comparing 2D_6_20 vs. 2D_6_60). In fact, the G_f_ does not directly reflect the actual crosslinking density, but rather the insoluble fraction, also potentially originating from crosslinking leading to pendant chains [43, 44, 45].

It is also interesting to notice how, the higher the crosslinking density, the lower is the degree of crystallinity. The χ_C_ vs. ν plot (Figure 7B) clearly illustrates this trend and can be used to describe structural variations within the system. The data also suggest that reversible, stress‐free shape‐memory behavior is associated with low χ_C_ and high ν values, corresponding to the bottom‐right region of the plot. However, the phenomenon is more complex than it appears, and a more detailed investigation would be necessary to fully elucidate the underlying mechanisms.

As evidenced by the previous discussion, it is clear that reversible, stress‐free shape‐memory behavior is achieved at high crosslinking temperatures. Nevertheless, these high temperatures are incompatible with good shape fidelity during printing. In fact, a low T_XL_ (T < T_c_) is convenient for 3D printing, as it allows for high shape fidelity during printing [29].

Nonetheless, we have here demonstrated that post‐treatment can be a valid solution to improve the overall performance of the specimens, especially in terms of two‐way shape‐memory performance. In this regard, the post‐treatment (Figure 7A) led to 2W‐SF values of 50%, midway between 2D_0.5_20 and 2D_6_60 (2W‐SF values of 0 and 100%, respectively).

In addition, to further increase the two‐way shape‐memory performance of the specimens, there is room for optimization of the thermal cycle parameters, in particular the ε_appl_ and the T_act_. In fact, increased values of initial applied strain (ε_appl_) have been reported to lead to higher AM and $\Delta \varepsilon_{rev(stress-free)}$ values [16, 29]. Moreover, high T_act_ was elsewhere reported to lead to increased $\Delta \varepsilon_{rev(stress-free)}$ in PCL‐based semi‐crystalline polymer networks [11, 35].

## 4 Conclusions

In this work, we investigated how the crosslinking parameters, namely the crosslinking temperature (T_XL_) and UV light intensity (I_XL_), influence the thermal, physical, mechanical, and shape‐memory properties of PCL‐based semi‐crystalline networks. We focused on crosslinking conditions achievable using a commercial printer, aiming to provide practical insights into the performance limits within this accessible printing window. We further compared 2D and 3D fabricated samples to evaluate the impact of the fabrication technique on material behavior. Overall, our findings demonstrate that the properties of the crosslinked networks can be effectively tuned by adjusting the crosslinking parameters. In fact, the obtained specimens exhibited χ_c_ values in the 29.1–36.9% range, G_f_ values in the 67.5–95.7% range, and E values in the 0.6 to 4.6 MPa range. Interestingly, the one‐way shape‐memory performance was only slightly affected by the crosslinking parameters or the fabrication process, having good performance under all the conditions explored. Conversely, both these latter factors significantly affected the two‐way shape‐memory behavior (both stress‐driven and stress‐free) of the samples. Notably, we showed that only crosslinking at elevated temperatures (T_XL_ > T_m_) enables stress‐free two‐way shape‐memory behavior, likely due to a combined effect involving the applied stress and the distribution of the crosslinking points within the three‐dimensional macromolecular network architecture. Last, we explored a post‐treatment strategy to enhance the functional properties of the samples, particularly their shape‐memory response. The post‐treatment was disclosed here as a highly effective approach, significantly improving the overall performance and enabling reversible actuation capabilities after fabrication.

In conclusion, this work offers practical guidelines for the optimal design of 2D and 3D structures made from PCL‐based semi‐crystalline networks with tailored physico‐mechanical, thermal, and shape‐memory performance.

## Conflicts of Interest

The authors declare no conflicts of interest.

## Data Availability

Data generated in this study have been deposited in the Zenodo database at https://doi.org/10.5281/zenodo.17119831
